# Abiotic synthesis of graphite in hydrothermal vents

**DOI:** 10.1038/s41467-019-13216-z

**Published:** 2019-11-15

**Authors:** Emily R. Estes, Debora Berti, Nicole R. Coffey, Michael F. Hochella, Andrew S. Wozniak, George W. Luther

**Affiliations:** 1 School of Marine Science and Policy, University of Delaware, 700 Pilottown Road, Lewes, DE 19958 USA; 2 Virginia Tech National Center for Earth and Environmental Nanotechnology (NanoEarth), 1991 Kraft Drive, Blacksburg, VA 24061 USA; 3 Subsurface Science and Technology Group, Energy and Environment Directorate, Pacific Northwest National Laboratory, Richland, WA 99352 USA; 4 Present Address: International Ocean Discovery Program, Texas A&M University, 1000 Discovery Drive, College Station, TX 77845 USA; 5 Present Address: Department of Oceanography, Texas A&M University, 3146 TAMU, College Station, TX 77843 USA

**Keywords:** Carbon cycle, Marine chemistry

## Abstract

Deciphering the origin, age, and composition of deep marine organic carbon remains a challenge in understanding the dynamics of the marine carbon cycle. In particular, the composition of aged organic carbon and what allows its persistence in the deep ocean and in sediment is unresolved. Here, we observe that both high and low temperature hydrothermal vents at the 9° 50′ N; 104° 17.5 W East Pacific Rise (EPR) vent field are a source for (sub)micron-sized graphite particles. We demonstrate that commonly applied analytical techniques for quantification of organic carbon detect graphite. These analyses thereby classify graphite as either dissolved or particulate organic carbon, depending on the particle size and filtration method, and overlook its relevance as a carbon source to the deep ocean. Settling velocity calculations indicate the potential for these (sub)micron particles to become entrained in the buoyant plume and distributed far from the vent fields. Thus, our observations provide direct evidence for hydrothermal vents acting as a source of old carbon to the deep ocean.

## Abstract (web summary)

Deciphering the origin, age, and composition of deep marine organic carbon remains a challenge for understanding the dynamics of the marine carbon cycle. Here, the authors identify (sub)micron-sized graphite emanating from both high and low temperature hydrothermal vents along the East Pacific Rise, and suggest graphite is a source of old carbon in the deep ocean.

## Introduction

Deep marine organic carbon is a mixture of fresh material delivered from the surface and an aged component, the origin of which is undetermined^1–4^. Chemically recalcitrant carbon, terrestrial black carbon, carbon from resuspended sediments, and hydrothermally derived carbon are considered possible sources of this fraction, yet their relative contributions and ages remain unconstrained^5–10^. In sediment, black carbon is substantially older than concurrently deposited sedimentary organic carbon^7,11^. The age offset is hypothesized to represent the residence time of black carbon in the reservoir of deep dissolved organic carbon (DOC), prior to sorption onto particulate organic carbon (POC) and deposition in sediment or, alternately, aging in terrestrial soils prior to mobilization and deposition in marine sediment^7^. Although the black carbon described in these previous studies is hypothesized to derive from the terrestrial combustion of biomass, additional evidence exists for a hydrothermal contribution to deep DOC and POC^2,6^ and to sedimentary black carbon^12^. DOC synthesized in hydrothermal systems is depleted in ^14^C, as it likely forms either from mantle-derived dissolved inorganic carbon or dissolved seawater CO_2_ aged during circulation of ridge flank fluids. This depletion is observed for both abiotically synthesized hydrocarbons^13^ and chemoautotrophic synthesis of DOC^6^. Here, we identify (sub)micron-sized graphite particles emanating from five different focused and low temperature hydrothermal vent sites at the EPR 9° 50′ N vent field, providing an alternate source of old carbon to the deep ocean reservoir of DOC and POC. Common analytical approaches to measuring DOC and POC also access graphite (Supplementary Tables 2 and 3). Thus, with the exception of the few studies directly targeting measurement of graphite, this portion of the deep sea carbon reservoir has been previously unrecognized and its contribution not quantified robustly.

## Results and discussion

### Hydrothermal graphite occurrence and morphology

**Fig. 1:**
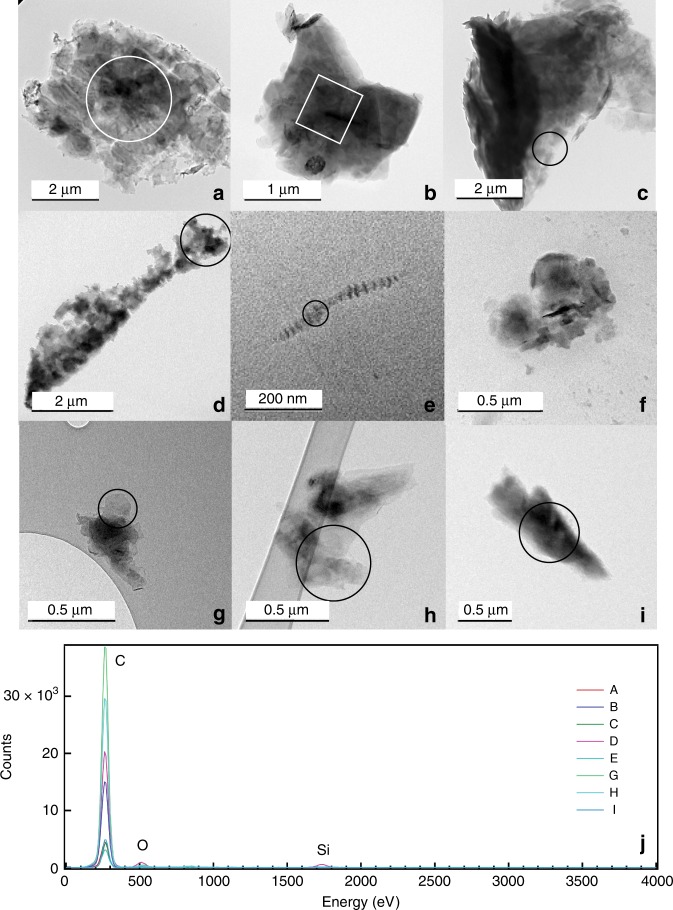
Transmission electron microscopy (TEM) images of graphitic carbon particles. Particles were identified in high-temperature fluids (Biovent, 335 °C, **a**, **b**), hydrothermal plumes (within 1 m of the vent orifice, Biovent, 15 °C, **c**–**f**, P Vent, 15 °C, **g**–**h**), and diffuse flow (Q Vent, 35 °C, **i**). Energy dispersive X-ray spectroscopy (EDS) spectra **j** taken from marked areas on particles show a common composition of carbon with trace oxygen and silicon, which likely represent background from the TEM grid and detector (Supplementary Information Fig. 1). No EDS spectrum was acquired from the particle in **f**

**Fig. 2:**
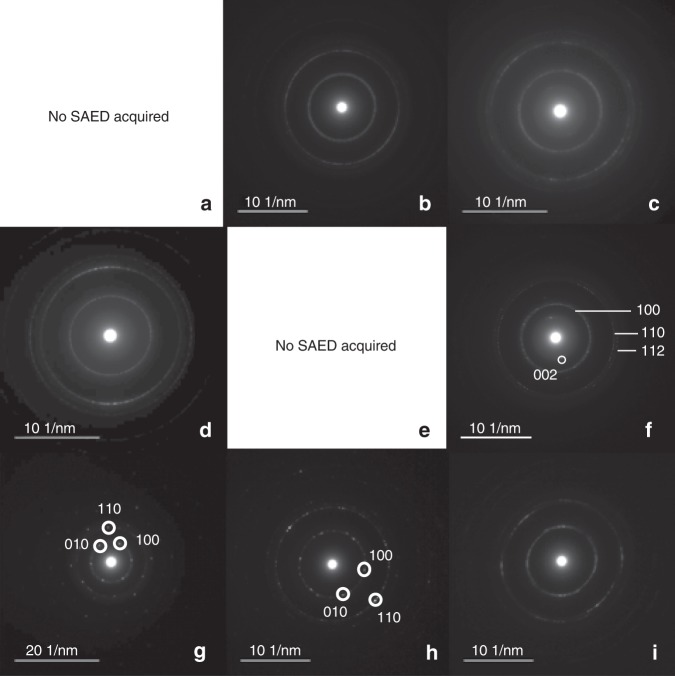
Selected area electron diffraction (SAED) patterns acquired from the particles shown in Figure 1. The ring patterns generated from most particles indicate either turbostratic disorder or platy graphite layers randomly rotated and translated with respect to each other. Particles f (Biovent, 15 °C) and g and h (P Vent, 15 °C) display symmetric spot patterns that match well with graphite along [001], as shown in the image

Particles were examined via transmission electron microscopy (TEM) and energy dispersive X-ray spectroscopy (EDS) (Table 1, Fig. 1). We found sub(micron) graphite in high-temperature end-member fluids and lower temperature samples in the hydrothermal plume within one meter of the chimney orifice, as well as in lower temperature vents (Table 1, Figs 1 and 2). Although we did not observe graphite in all samples, the absence of detection does not indicate that a vent was not emitting this material. Our TEM approach did not target detection and identification of graphite. Graphite was not observed in the end-member fluids (361 °C) captured at P Vent, but was found in an ~ 15 °C sample taken from higher in the hydrothermal plume (Fig. 1) at that site. At Biovent, graphite was observed in the end-member fluids (335 °C) and in a 15 °C plume sample, but not in a 185 °C sample from mid-height in the plume. One of the two samples analyzed from the low temperature Q Vent (72 °C) contained graphite; graphite was not observed in the sample from Tica, a diffuse flow site. Overall, graphite was observed in 4 of the 10 samples examined (Table 1) and accounted for 6% of the area in 84 images of particles with known composition (including the more prevalent sulfide and silicate particles).

**Table 1:** Index of all graphitic particles identified and characterized via transmission electron microscopy

ID	Vent	Sample type	Temperature (°C)	Latitude	Longitude	Fe (II) (μm)	Total reduced S (μm)	Longest dimension (μm)	Stokes settling velocity (m yr^−1^)
A	Biovent	End-member	335	9° 50.96′ N	104° 17.62′ W	338 ± 3.05	5170 ± 855	6.55	470
B	Biovent	End-member	335	9° 50.96′ N	104° 17.62′ W	338 ± 3.05	5170 ± 855	2.40	63.2
C	Biovent	Plume	15	9° 50.97′ N	104° 17.62′ W	7.80 ± 0.19	159 ± 29.2	7.19	567
D	Biovent	Plume	15	9° 50.97′ N	104° 17.62′ W	7.80 ± 0.19	159 ± 29.2	6.46	458
E	Biovent	Plume	15	9° 50.97′ N	104° 17.62′ W	7.80 ± 0.19	159 ± 29.2	0.35	1.35
F	Biovent	Plume	15	9° 50.97′ N	104° 17.62′ W	7.80 ± 0.19	159 ± 29.2	0.78	6.74
G	P vent	Plume	15	9° 50.28′ N	104° 17.47′ W	36.3 ± 0.35	42.2 ± 14.4	0.59	3.84
H	P vent	Plume	15	9° 50.28′ N	104° 17.47′ W	36.3 ± 0.35	42.2 ± 14.4	1.05	12.1
I	Q vent	Low °T	35	9° 50.73′ N	104° 17.59′ W	393 ± 5.05	249 ± 49.0	1.40	21.5

Sample location as well as iron (Fe) and reduced sulfur (S) (average and standard deviation of three analyses) in unfiltered fluids are provided for geochemical context (see methods). All Fe was measured as Fe(II), either dissolved or present in sulfide phases; statistically significant concentrations of Fe(III) were not detected. A maximum estimated Stokes settling velocity for each particle was calculated from the longest measured dimension

The majority of particles appear as aggregates of sheet-like structures with irregular edges (Fig. 1). This morphology matches the previous observation of graphite in sediment traps designed to collect fallout of vent material, suspended 50 m above the seafloor at distances of 200–300 m from the vent source at EPR 13° N^8^. This graphite was hypothesized to be hydrothermally derived and likewise consisted of large angular fragments overgrown with smaller platelets^8^. Exceptions in our samples include particle D (Biovent plume, 15 °C), which appears to be a greater number of small, aggregated fragments, and particle E (Biovent plume, 15 °C), a long thread-like particle with alternating regions of high and low electron density (Fig. 1). Although our image is not well-resolved, this morphology is similar to polygonal graphite grains previously observed in metasediment and carbonate veins^14^. Rough particle edges and surfaces in the TEM images (Fig. 1) likely reflect micro- and nano-crystals not resolved at this magnification. Particle size ranges from 350 nm to 7.2 microns along the longest dimension (Table 1), smaller than the average particle size of 15 microns observed in the sediment traps deployed at the EPR 13° N vent field^8^. This size discrepancy may indicate that the smaller particles we observe are dispersed further from the vent site than the 200–300 m distances used in the previous work^8^. As we observe no larger particles, we posit that particles in this < 10 micron size fraction account for the majority of the graphite flux and that this flux has been underestimated. It is possible although less likely that continued aggregation and growth during transport generates the larger particles observed previously.

### Chemical composition and structure

All particles had near-identical chemical compositions, consisting of carbon with very small amounts of oxygen, silicon, and nickel. All of these elements are likewise present in the TEM grid/blank; whereas nickel derives from the grid, there could be trace oxygen and silicon in the samples as well as the blanks (Fig. 1, Supplementary Fig. 1). In addition to EDS analysis, selected area electron diffraction (SAED) patterns were collected from seven of the nine particles to test for particle crystallinity (Fig. 2). Graphite sheets/layers tend to orient on the TEM grid, resulting in SAED patterns that only show reflections from (hk0) crystal planes. Therefore, for some particles (B, C, D, G), multiple SAED patterns were acquired after tilting the sample stage. Identification was further confirmed by high resolution TEM images (HRTEM) taken from particles edges (Supplementary Fig. 2). Measurements from SAED patterns and HRTEM match the d-spacings of graphite (American Mineralogist Crystal Structure Database, amcsd 14675) planes, varying from the AMCSD d-spacings by < 2.5% in all instances (Supplementary Table 1 and Fig. 2). The presence of spots and rings in all observed diffraction patterns indicates a mixture of both highly crystalline graphite and graphite layers randomly rotated and translated with respect to each other. We could not resolve whether the layers represent ultrathin single crystals with turbostratic disorder (compare with ref. ^15,16^) or multiple, platy crystals stacked with differing planar orientations (compare with ref. ^16^). However, the 002 reflection in some of the oriented SAED patterns is explained by curled edges visible in TEM and HRTEM images. Further, the close correspondence in d-spacing between our samples and the AMCSD pattern suggests disorder is primarily planar rather than turbostratic, the latter of which would result in increased d-spacings^15,16^. In particular, the broad and diffuse rings of particles B, C, D, and F reflect small crystal size on the order of 3–8 nm in addition to much larger crystals that generate the diffraction spots within the rings (Fig. 2). One SAED pattern acquired from an extremely thin region of particle G shows a symmetric spot pattern matching the graphite 001 zone axis; faint rings from less ordered, smaller crystals are visible underneath (Fig. 2).

### Transport potential and implications for deep sea carbon

To estimate transport potential, we utilized the longest dimension of these irregular-shaped particles as the radius and assumed the density of graphite (2.23 g cm^−3^) to calculate Stokes settling velocities ranging between 1.35 and 567 m yr^−1^ (Table 1). Less ordered or turbostratic graphite may be less dense and particles are flat and platy rather than spherical; consequently, our estimates of settling velocity are conservatively high. Compared with the 15 micron diameter sediment trap particles collected near the 13° N EPR vent field^8^, we expect that the smaller particles we observe in vent fluids at 9° 50′ N EPR will be entrained higher in the rising plume and transported significantly greater distances. Dissolved iron derived from hydrothermal vents along the southern EPR is transported distances > 4000 km from the vent source, likely stabilized by organic ligands^17^. The contribution of nanoparticulate phases to the operationally dissolved portion of hydrothermal plumes has not yet been quantified, although nanoparticulate pyrite is kinetically stable against oxidation over the timescales of transport^18^. Pyrite nanoparticles with a 200 nm diameter have a Stokes settling velocity of 1.43 m/yr^18^; comparable to the 1.35 m/yr calculated for our smallest observed graphite particle. At the EPR 9° 50′ N vent field, seasonal deep-reaching mesoscale eddies may generate current velocities an order of magnitude higher than those in the southern EPR, up to 15 cm/s^19^. Following this logic, we posit that hydrothermal graphite particles could disperse thousands of kilometers from the vent site.

Accordingly, H/C vs O/C van Krevelen plots identify carbon-rich molecules that make up a small fraction of the deep Pacific DOC^20^ that could represent graphite or graphite altered by reactions with reactive oxygen species in the vent mixing zones. Circulation of seawater through basalt in the Juan de Fuca Ridge Flank results in an overall removal of DOC but the addition of aromatic compounds^21^, similar to the high aromaticity observed in fluids collected along the Mid-Atlantic Ridge^22^. These aromatics may derive from hydrothermal alteration of organic matter^23,24^ or, in part, graphite alteration reactions. Further, over half of the black carbon in an Equatorial Pacific sediment sample at 8° 55′ N and 139° 52′ W (~ 3900 km from our EPR site, but closer to other vent sites) consisted of either impure or highly organized graphite^12^, such as that observed here. The study describing this sedimentary graphite discounted a hydrothermal source given the distance and lack of metalliferous sediment; however, here, we characterize particles with the requisite transport potential, as determined from the Stokes settling velocity calculations. These particles may sink at distance from the vent source and not co-locate with metal enrichments.

### Formation pathways

Graphite is known to form under high-temperature/high pressure conditions both from the reduction of CO or CO_2_ and graphitization of organic matter yet it has not previously been considered as a component of black smoker fluids. Here, we suggest that micron- and submicron-sized particles form in venting hot hydrothermal fluids or become mobilized from deeper deposits. Indeed, thermodynamic calculations indicate that graphite will form upon conductive cooling of representative hydrothermal end-member fluids^25^. The reaction pathway leading to the formation of hydrothermal graphite depends on the system geochemistry, but commonly proceeds via catalytic disproportionation of CO or reduction of CO and CO_2_ as described by Jedwab and Boulègue^8^ and Mathez and Delaney^26^:

1 $$2{\mathrm{CO}} \to {\mathrm{C}} + {\mathrm{CO}}_2\left( {{\mathrm{catalyzed}}\,{\mathrm{by}}\,{\mathrm{metal}}\,{\mathrm{sulfides}}} \right)$$

2 $${\mathrm{CO + H}}_2 \to {\mathrm{C + H}}_2{\mathrm{O}}\left( {{\mathrm{catalyzed}}\,{\mathrm{by}}\,{\mathrm{iron}}} \right)$$

3 $${\mathrm{CO}}_2 \to {\mathrm{C + O}}_2$$

Under the temperature and pressure regimes found in the vent systems considered here, CO_2_ will predominate over CO^26^. As concentrations of Fe and sulfide are elevated in hydrothermal fluids (mm), these species may nonetheless catalyze reactions of the trace concentrations of CO. In basalts, elemental carbon phases occur along quench-produced microcracks, associated with metal sulfide spherules along the edges of vesicles, and in bubble inclusions^26^. These observed associations in conjunction with the abundance of Fe and sulfide in hydrothermal fluids implicate Eqs. (1) and (2) as likely reaction pathways although we do not discount Eq. (3). Graphitization of organic matter is another possible pathway for formation, although this reaction may require higher temperatures and pressures than these vent samples would have experienced as well as an initial carbonization step; the process is typically considered to require metamorphism^27^. In laboratory settings, however, iron^28^ and base metal (Ni, Fe, and Co) nanoparticles^29^ can catalyze graphitization and may likewise do so in the vent environment. Combustion or pyrolysis reactions, likewise, may produce black carbon and biochar compounds consisting of poorly ordered graphene and turbostratic graphite^30^. We observe several particles primarily composed of carbon but containing minor concentrations of nitrogen and oxygen that may be combustion or pyrolytic residues, representing organic matter undergoing alteration to graphite (Supplementary Fig. 3). These pathways are interesting targets for future investigations.

We demonstrate the presence of graphite in a range of hydrothermal fluids and suggest that vents globally are a source of graphite. Our observations thereby provide evidence for a significant but unconstrained flux of (sub)micron graphite to the ocean and sediment reservoirs of carbon. We demonstrate that this fraction may be misclassified as DOC or POC using common analytical techniques (Supplementary Tables 2 and 3) and emphasize the need for targeted measurements. As this hydrothermal graphite likely forms from mantle-derived carbon, we expect the particles to be highly ^14^C depleted or radiocarbon-dead. These newly described (sub)micron-sized particles therefore confirm hydrothermal vents as a source of old carbon that can help explain age discrepancies in deep ocean DOC^2^ and sedimentary carbon^7^.

## Methods

### Samples and sample processing

Samples of vent fluids were taken using the Human Operated Vehicle Alvin II and the titanium Major Samplers at multiple focused, low temperature, and diffuse flow sites along EPR 9° 50′ N vent field. Sample temperature was recorded using the ICL high-temperature probe attached to the Major Sampler. Samples were processed immediately shipboard, ~ 2–4 h after collection. For each microscopy sample, between 20–80 mL of fluid was syringe-filtered onto a 0.2 μm polycarbonate filter, rinsed with de-aerated 18 MΩ water, and dried under Ar. Volume filtered was a function of particle content; particle-rich fluids often clogged filters after 20 mL had passed through. Samples were preserved under Ar until analysis.

### TEM and SAED

Particles on the polycarbonate filters were resuspended in distilled water by sonication. A drop of suspension was deposited on ultrathin C or lacey C supported nickel TEM grids and allowed to dry. The grids were analyzed in a JEOL JEM-2100 analytical TEM, operated at 200 keV. This TEM is equipped with a 60 mm^2^ window, JEOL EX-230 Silicon Drift Detector (SDD) that allows acquisition of EDS spectra with a spatial resolution of 3 nm in STEM mode, and in the range of 5–10’s of nanometers in parallel illumination TEM mode. Images were acquired at different magnifications to characterize morphology and size of particles. SAED patterns were acquired from representative particles and analyzed using Gatan Digital Micrograph software (GMS 3) to measure crystalline features. EDS spectra were collected from the same regions. Minerals were identified based on both EDS spectra and SAED patterns.

### Dissolved carbon analyses

To prepare a graphite dissolved carbon standard, 0.0665 g of graphite (Ward’s Science) was suspended in 1 L of filtered seawater (FSW) collected offshore of Delaware. Homogeneity and particle disaggregation were ensured by sonication. In triplicate, 60 mL of the graphite/FSW mixture were vacuum filtered using pre-combusted GF/F filters (Whatman, 0.7 μm nominal pore size). Filtrate was immediately poured into 30 mL vials, acidified using 2 m HCl, and analyzed on a Shimadzu TOC-V CPH Total Organic Carbon Analyzer. The instrument was operated at 680 °C using UHP air as the carrier gas and calibrated using a five-point standard curve created with a potassium hydrogen phthalate (LabChem) standard. Concentrations were determined using the best three of five injections per sample. Acidified FSW samples (no graphite added) were analyzed in the same way and in the presence of pre-combusted glass beads, and graphite suspended samples were compared with these control samples to determine whether graphite was detected in the operationally-defined DOC. The difference in DOC concentrations between FSW and FSW with glass bead samples was not statistically different than 0 (0.6 ± 4 µm; *n* = 4). A Deep Sea Reference (Hansell Laboratory, DSR Lot # 07–07, 41–45 μm DOC) was also run as a quality control. A value of 47.8 ± 2.0 μm, *n* = 3 was measured for this reference standard.

### Particulate carbon analyses

Graphite (Ward’s Science) standard was dried in an oven at 54 °C for 24 h and stored in a desiccator until analysis. The graphite was weighed into tin capsules (Elemental Microanalysis). NIST Standard Reference Material 2975 Diesel Particulate Matter (Industrial Forklift, reported previously as 84 ± 9% OC^25^ and 80 ± 14% OC^26^) was prepared in the same manner as a quality control. These samples were then analyzed on a Costech ECS 4010 Elemental Combustion System CHNS-O operated in CHN mode with two furnaces. The first furnace contained a chromium oxide and silver cobaltous/cobaltic oxide column held at 980 °C; the second furnace contains a reduced copper wire column and is held at 650 °C. He was used as a carrier gas (100 mL/min) and O_2_ flowrate was 25 mL/min. The instrument was calibrated using an eight-point calibration curve created using both reagent-grade phenylalanine (Sigma Aldrich) and ethylenediaminetetraacetic acid (Sigma Aldrich) standards. SRM-2975 was measured as 81.5 ± 7.1% OC, *n* = 3, in agreement with previously published analyses^31,32^.

### Stokes settling velocities

Maximum Stokes settling velocities were calculated from the longest particle dimension and a density of 2.23 g cm^−3^, assuming a spherical particle and following Yücel et al.^18^ and Schwarzenbach et al.^33^. We consider this calculation an overestimate as particles are not spherical and are likely less dense if less well-ordered than fully crystalline graphite. Particle dimensions were determined using the Fiji software package^34^. Under laminar flow, the equation is written as:

$${\mathit{V}} = {\mathit{g}} \ast D^2 \ast \left( {d_p-d_m} \right)/\left( {18 \ast v} \right)$$

*V*: settling velocity (m s^−1^)

*g*: gravitational acceleration, 9.8 m s^−2^

*D*: particle diameter (m)

*d*_p_: particle density, 2230 kg m^−3^ for graphite

*d*_m_: medium density, 1029 kg m^−3^ for seawater

*v*: viscosity of seawater, 1.88 × 10^−3^ kg m^−1^ s^−1^

### Iron and sulfur concentrations

Total iron and reduced sulfur in unfiltered fluids were analyzed spectrophotometrically using the ferrozine and acid volatile sulfides/chromium-reducible sulfides (AVS/CRS) methods, respectively. These methods are described in Findlay et al.^35^.

## Supplementary information

Supplementary Information

## Data Availability

TEM images, SAED patterns, and EDS data are available from the authors upon request.

## References

[1] Hansell DA (2013). Recalcitrant dissolved organic carbon fractions. Ann. Rev. Mar. Sci..

[2] Druffel ERM, Griffin S (2015). Radiocarbon in dissolved organic carbon of the South Pacific Ocean. Geophys. Res. Lett..

[3] Walker BD, Beaupré SR, Guilderson TP, McCarthy MD, Druffel ERM (2016). Pacific carbon cycling constrained by organic matter size, age and composition relationships. Nat. Geosci..

[4] Williams PM, Druffel ERM (1987). Radiocarbon in dissolved organic matter in the central North Pacific Ocean. Nature.

[5] Jiao N (2010). Microbial production of recalcitrant dissolved organic matter: long-term carbon storage in the global ocean. Nat. Rev. Microbiol..

[6] McCarthy MD (2011). Chemosynthetic origin of 14C-depleted dissolved organic matter in a ridge-flank hydrothermal system. Nat. Geosci..

[7] Coppola AI, Ziolkowski LA, Masiello CA, Druffel ERM (2014). Aged black carbon in marine sediments and sinking particles. Geophys. Res. Lett..

[8] Jedwab J, Boulègue J (1984). Graphite crystals in hydrothermal vents. Nature.

[9] Hansell D, Carlson C, Repeta D, Schlitzer R (2009). Dissolved organic matter in the ocean: a controversy stimulates new insights. Oceanography.

[10] Beaupré SR, Druffel ERM (2009). Constraining the propagation of bomb-radiocarbon through the dissolved organic carbon (DOC) pool in the northeast Pacific Ocean. Deep Sea Res. Part I Oceanogr. Res. Pap..

[11] Masiello CA, Druffel ERM (1998). Black carbon in deep-sea sediments. Science.

[12] Haberstroh PR (2006). Chemical composition of the graphitic black carbon fraction in riverine and marine sediments at sub-micron scales using carbon X-ray spectromicroscopy. Geochim. Cosmochim. Acta.

[13] Proskurowski G (2008). Abiogenic hydrocarbon production at lost city hydrothermal field. Science.

[14] Ohtomo Y, Kakegawa T, Ishida A, Nagase T, Rosing MT (2014). Evidence for biogenic graphite in early Archaean Isua metasedimentary rocks. Nat. Geosci..

[15] Binder C (2017). Structure and properties of in situ-generated two-dimensional turbostratic graphite nodules. Carbon N. Y..

[16] Groopman E, Nittler LR, Bernatowicz T, Zinner E (2014). NanoSIMS, TEM, and XANES studies of a unique presolar supernova graphite grain. Astrophys. J..

[17] Resing JA (2015). Basin-scale transport of hydrothermal dissolved metals across the South Pacific Ocean. Nature.

[18] Yücel M, Gartman A, Chan CS, Luther GW (2011). Hydrothermal vents as a kinetically stable source of iron-sulphide-bearing nanoparticles to the ocean. Nat. Geosci..

[19] Adams DK (2011). Surface-generated mesoscale eddies. Science.

[20] Bercovici SK (2018). Aging and molecular changes of dissolved organic matter between two deep oceanic end-members. Glob. Biogeochem. Cycles.

[21] Lin HT, Repeta DJ, Xu L, Rappé MS (2019). Dissolved organic carbon in basalt-hosted deep subseafloor fluids of the Juan de Fuca Ridge flank. Earth. Planet. Sci. Lett..

[22] Rossel PE (2017). Thermally altered marine dissolved organic matter in hydrothermal fluids. Org. Geochem..

[23] Simoneit BRT (1993). Aqueous high-temperature and high-pressure organic geochemistry of hydrothermal vent systems. Geochim. Cosmochim. Acta.

[24] Dittmar T, Koch BP (2006). Thermogenic organic matter dissolved in the abyssal ocean. Mar. Chem..

[25] Bowers TS, Von Damm KL, Edmond JM (1985). Chemical evolution of mid-ocean ridge hot springs. Geochim. Cosmochim. Acta.

[26] Mathez EA, Delaney JR (1981). The nature and distribution of carbon in submarine basalts and peridotite nodules. Earth. Planet. Sci. Lett..

[27] Buseck PR, Beyssac O (2014). From organic matter to graphite: graphitization. Elements.

[28] Thompson. E, Danks AE, Bourgeois L, Schnepp Z (2015). Iron-catalyzed graphitization of biomass. Green. Chem..

[29] Hoekstra J (2015). Base metal catalyzed graphitization of cellulose: a combined Raman spectroscopy, temperature-dependent X-ray diffraction and high-resolution transmission electron microscopy study. J. Phys. Chem. C..

[30] Keiluweit M, Nico PS, Johnson MG, Kleber M (2010). Dynamic molecular structure of plant biomass-derived black carbon (biochar). Environ. Sci. Technol..

[31] Elmquist, M. et al. Distinct oxidative stabilities of char versus soot black carbon: Implications for quantification and environmental recalcitrance. *Global Biogeochem. Cycles*. **20****:** 0.1029/2005GB002629 (2006).

[32] Han Y (2007). Evaluation of the thermal/optical reflectance method for discrimination between char- and soot-EC. Chemosphere.

[33] Schwarzenbach, R. P., Gschwend, P. M. & Imboden, D. M. *Environmental Organic Chemistry*. (John Wiley and Sons, Inc. 1993).

[34] Schindelin J (2012). Fiji: An open-source platform for biological-image analysis. Nat. Methods.

[35] Findlay, A. J., Estes, E. R., Gartman, A., Yucel, M., Kamyshny Jr., A., & Luther III, G. W. Iron and sulfide nanoparticle formation and transport in nascent hydrothermal vent plumes. *Nat. Commun.* **10**, 1597 (2019). 10.1038/s41467-019-09580-5 PMC6453976 30962453

