# Supplementary material for: Abiotic synthesis of graphite in hydrothermal vents
Source: Nat Commun. 2019 Nov 15;10:5179. doi: 10.1038/s41467-019-13216-z (PMC6858401; doi:10.1038/s41467-019-13216-z)
Supplement: Supplementary file 1 — Supplementary Information [file 41467_2019_13216_MOESM1_ESM.pdf]

Abiotic synthesis of graphite in hydrothermal vents

Supplementary Information

Emily R. Estes[1,4*], Debora Berti[2,5], Nicole R. Coffey[1], Michael F. Hochella Jr.[2,3], Andrew S. Wozniak[1], and George W. Luther III[1]

1. University of Delaware, School of Marine Science and Policy, 700 Pilottown Road, Lewes, DE 19958 USA
2. Virginia Tech National Center of Earth and Environmental Nanotechnology Infrastructure (NanoEarth), Institute for Critical Technology and Applied Science, Virginia Tech, Blacksburg, VA 24061 USA
3. Subsurface Science and Technology Group, Energy and Environment Directorate, Pacific Northwest National Laboratory, Richland, WA 99352 USA
4. *now at* International Ocean Discovery Program, Texas A&M University, 1000 Discovery Drive, College Station, TX, 77845 USA, estes@iodp.tamu.edu
5. *now at* Texas A&M University, Department of Oceanography, 3146 TAMU, College Station, TX 77843 USA

*corresponding author

Supplementary Figure 1. (A) EDS spectra acquired from five blank regions on the TEM grids. Spectra show that carbon, oxygen, silicon, and nickel are the major elements detected in the blank. (B) The ratio of C:Ni intensity in the blank samples is 5.2 ± 0.8, (C) while samples have C:Ni values of between 7.6 (Biovent, 15 °C, particle E) and 70 (Biovent, 15 °C, particle C), indicating that carbon observed in the analyzed particles derives from the particles and not the TEM grid or another artifact. The one exception is particle A (Biovent, 335 °C) which has a C:Ni of 2.3; it is unclear why signal from the TEM grid predominates in this sample but note that compositionally it also appears to be pure carbon.

A

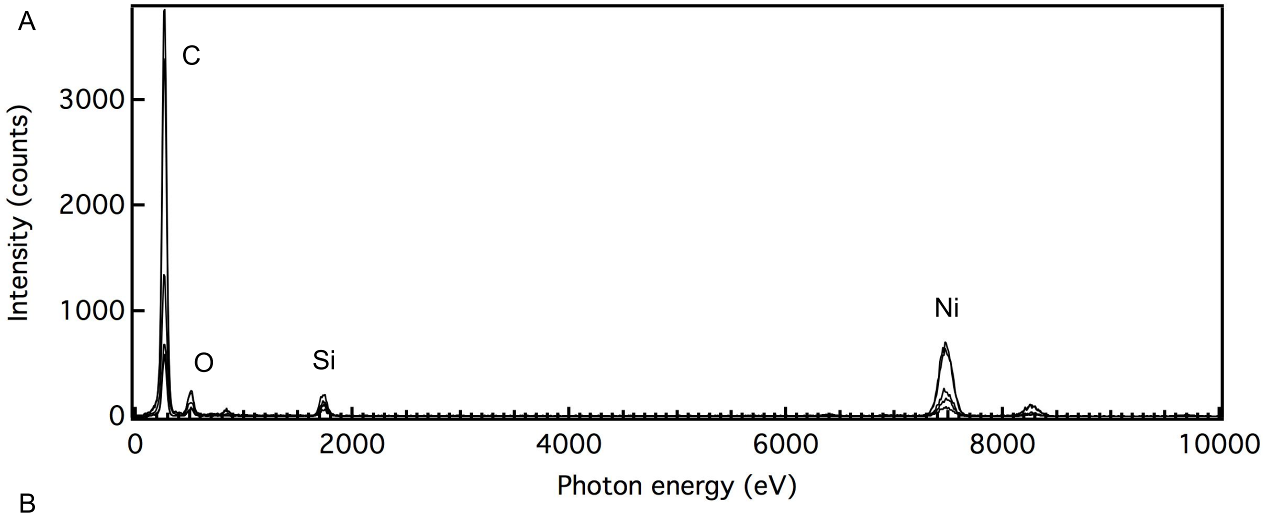

B

blank	C counts	Ni counts	C/Ni
1	1344	266	5.05
2	589	93	6.33
3	683	172	3.97
4	3839	706	5.44
5	3394	644	5.27

C

#	vent	sample type	sample temperature (°C)	C counts	Ni counts	C/Ni
A	Biovent	end-member	335	4576	1951	2.3
B	Biovent	end-member	335	15082	373	40.4
C	Biovent	plume	15	4348	62	70.1
D	Biovent	plume	15	20376	1114	18.3
E	Biovent	plume	15	3088	409	7.6
F	P Vent	plume	15	38726	1389	27.9
G	P Vent	plume	15	29679	922	32.2
H	Q Vent	low-temperature	35	4921	99	49.7

Supplementary Table 1. Measured d-spacings (Å) from SAED patterns and from HRTEM images compared with those from the American Mineralogist Crystal Structure Database entry 14675. Blank spaces indicate that a ring or spot corresponding to that reflection was not observed.

	Particle							
**hkl**	**B**	**C**	**D**	**F**	**G**	**H**	**I**	**AMCSD 14675**
002	3.36	3.36	3.35	3.38	3.35			3.36
100	2.15	2.08	2.11	2.08	2.12	2.17	2.14	2.13
101	2.04				2.06		2.03	2.03
110	1.24	1.24	1.23	1.22	1.24	1.26	1.24	1.23
112	1.16	1.15		1.16	1.16	1.18	1.15	1.16
200	1.07	1.06	1.07		1.06	1.09		1.07

Supplementary Figure 2. Additional selected area diffraction patterns and high resolution TEM images from particles B, C, D, and G, with diagnostic reflections and d-spacings indicated.

Particle B:

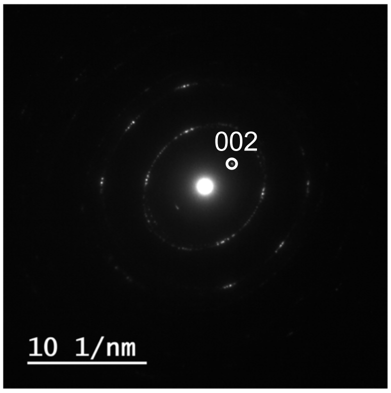

SAED, spot from the 002 plane with characteristic 3.36 Å d-spacing marked

Particle C:

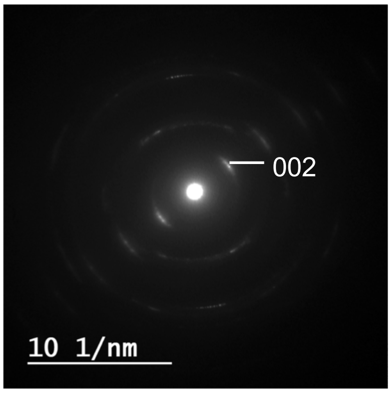

SAED, ring from the 002 plane with characteristic 3.36 Å d-spacing marked

Particle D:

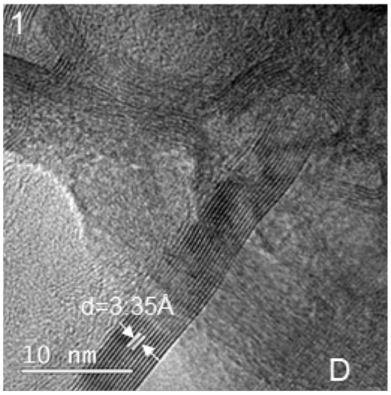

HRTEM with 3.35 Å d-spacing marked

Particle G:

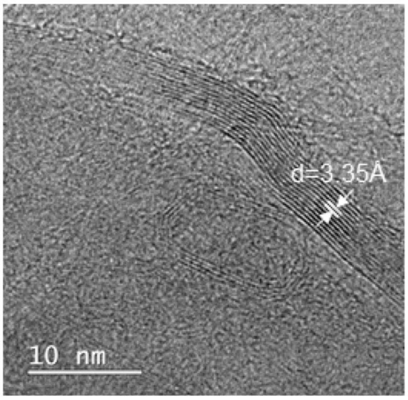

HRTEM with 3.35 Å d-spacing marked

Supplementary Figure 3. TEM images of carbon-rich, nitrogen-bearing particles that may reflect organic matter undergoing thermal alteration.

Q vent, 72° C fluid

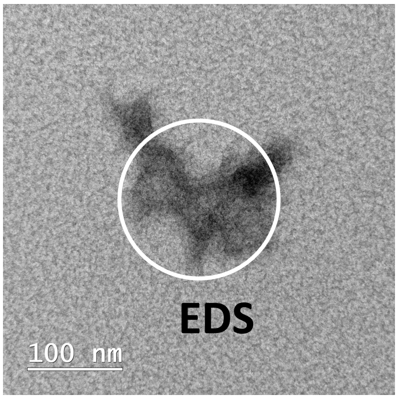

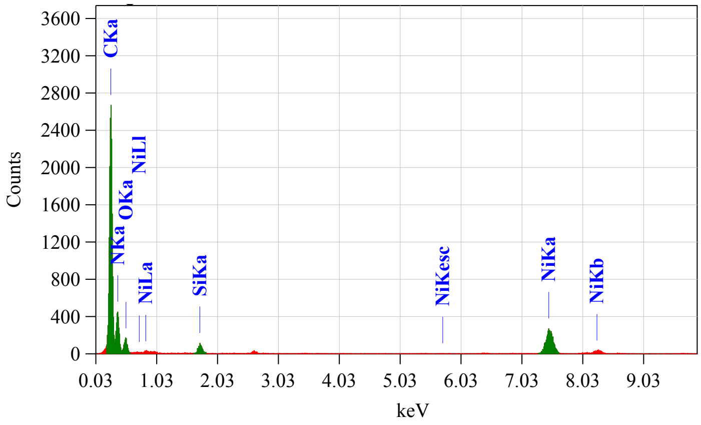

Biovent, 15° C fluid

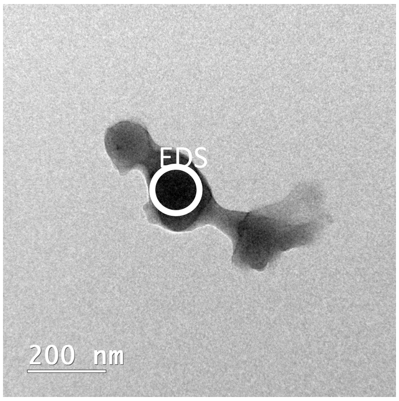

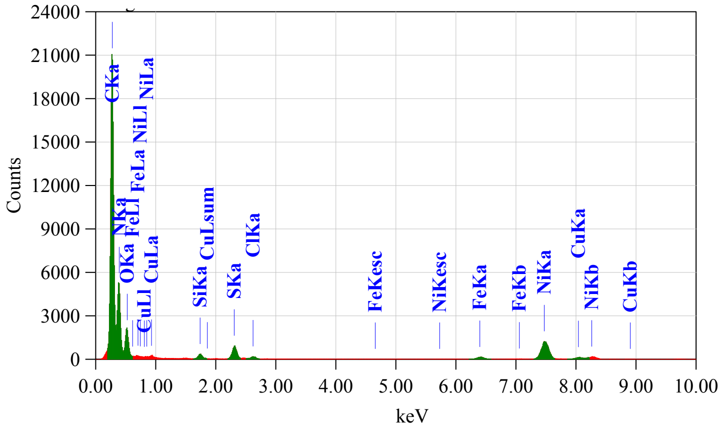

Supplementary Table 2. Results from dissolved organic carbon analysis of filtered seawater (FSW) and seawater spiked with graphite and re-filtered. Samples spiked with graphite show an increase in measured carbon, where graphite detected represents the difference between the spiked sample and the FSW sample. This result demonstrates detection of graphite by this commonly applied technique. Note that percent recoveries are low as the majority of the graphite particles in the standard were larger than the pore size of the GF/F filter and were removed from solution. Error is the standard deviation of the mean of three injections.

Sample	DOC (μM)	graphite detected (μM)	% of total graphite added
FSW	251.1 ± 2.5	--	--
graphite-spiked FSW replicate 1	325.5 ± 3.5	74.4	1.3
graphite-spiked FSW replicate 2	305.2 ± 2.2	54.1	1.0
graphite-spiked FSW replicate 3	329.8 ± 7.5	78.7	1.4

Supplementary Table 3. Results from Elemental Analysis of diesel particulate matter and graphite standards, demonstrating that particulate graphite can be quantitatively measured via EA and may be misclassified as a component of particulate organic carbon.

Sample	n	% recovery	expected % recovery
diesel particulate matter	3	81.5 ± 7.1	80
graphite	2	103.5 ± 0.6	100
